# A review of selected research priority setting processes at national level in low and middle income countries: towards fair and legitimate priority setting

**DOI:** 10.1186/1478-4505-9-19

**Published:** 2011-05-15

**Authors:** Mark Tomlinson, Micky Chopra, Naeema Hoosain, Igor Rudan

**Affiliations:** 1 Department of Psychology, Stellenbosch University, South Africa; 2 UNICEF, New York, USA; 3 Health Systems Research Unit, Medical Research Council, South Africa; 4 Croatian Centre for Global Health, University of Split Medical School, Croatia; 5 Centre for Population Health Sciences, University of Edinburgh Medical School, UK

## Abstract

**Background:**

It is estimated that more than $130 billion is invested globally into health research each year. Increasingly, there is a need to set priorities in health research investments in a fair and legitimate way, using a sound and transparent methodology. In this paper we review selected priority setting processes at national level in low and middle income countries. We outline a set of criteria to assess the process of research priority setting and use these to describe and evaluate priority setting exercises that have taken place at country level. Based on these insights, recommendations are made regarding the constituents of a good priority setting process.

**Methods:**

Data were gathered from presentations at a meeting held at the World Health Organization (WHO) in 2008 and a web-based search. Based on this literature review a number of criteria were developed to evaluate the priority setting processes.

**Results:**

Across the countries surveyed there was a relative lack of genuine stakeholder engagement; countries varied markedly in the extent to which the priority setting processes were documented; none of the countries surveyed had a systematic or operational appeals process for outlined priorities; and in all countries (except South Africa) the priorities that were outlined described broad disease categories rather than specific research questions.

**Conclusions:**

Country level priority setting processes differed significantly in terms of the methods used. We argue that priority setting processes must have in-built mechanisms for publicizing results, effective procedures to enforce decisions as well as processes to ensure that the revision of priorities happens in practice.

## Background

It is estimated that more than $130 billion is invested globally into health research each year, and the amount has been increasing steadily over the past decade [1]. In spite of this, request for funds far exceeds available resources. We have described elsewhere how a relative lack of transparency, accountability to high-level goals and strategic directions, and difficulties in determining where particular research fits in the process of knowledge translation have led to significant imbalances in investing [1].

Increasingly, there is a need to set priorities in health research investments in a fair and legitimate way, using a sound and transparent methodology. Research can play a critical role in the response to global health challenges, but with limited resources guidelines are needed to assist defining the priorities for health research investments. The explicit and rational setting of priorities for investment in research is now accepted as an integral part of any research management process [2] National research priority setting, if it includes a regional and international perspective can also feed into and drive regional and international agendas rather than 'respond' to the agendas suggested by others. A number of countries have embarked upon explicit national health research priority exercises but there has been little systematic assessment and analysis of the processes followed or the outcomes.

In 2008, a workshop on Priority Setting Methodologies in Health Research was held at the World Health Organization (WHO) in Geneva. The workshop objective was to develop practical proposals for user friendly methodologies for priority setting in health research for application in low and middle income countries. In this paper we review current research priority setting processes at national level in selected low and middle income countries. We outline a set of criteria to assess the process of research priority setting, use these to describe and evaluate priority setting exercises that have taken place at country level, and finally conclude with a discussion of the ingredients of a good priority setting process

### Establishing criteria to assess research priority setting initiatives

At its core, priority setting involves adjudicating between different dimensions that require understanding and application of specific values. Some of the dimensions which conflict include: benefit, evidence, cost, efficiency, equity, equality, benefit to a country's economy, severity of disease, prevalence of disease, solidarity, protection of the vulnerable, and more. In a pluralistic society we will never have complete agreement about what decisions to make, or about what outcomes are correct or preferable. As a result, an ethical framework that emphasizes the process by which priority setting occurs is needed, rather than an outlining of simple rules or outcomes [4]. The focus of an ethical priority setting process then becomes one of a focus on fair process and a shift away from what decisions are made to how decisions are made [5]. According to Daniels and Sabin [6] the extent to which it is clear how decisions were arrived at *(transparency) *is crucial.

Another key feature in such contexts is the legitimacy of those who establish and participate in the priority setting process. *Legitimacy *refers to the moral authority of decision makers (what are the conditions for when a group, organization or person should be given the task of setting priorities), while *fairness *refers to the moral acceptability (when is there sufficient reason to accept that the decision made are fair) of the decision making process [6,7]. Since the specific value drivers for health research prioritization may vary depending upon the context, these prioritization decisions must be made at the country level, rather than at a global level. The elements of legitimate and fair priority setting should provide a guiding framework for developing a decision making process and methodology. Martin [3] has stated that the guiding principles in this process should be one of inclusivity in order to ensure as wide participation as possible.

In terms of legitimacy and fairness Bruni and colleagues have argued that the public is the most important stakeholder in the health care system [8]. Following Kapiriri and colleagues, the term "stakeholders" refers to all individuals and/or groups who have an interest in the prioritization of health investments and could thus be a large and heterogeneous group [9]. But the involvement of the public must be genuine and they must not be left feeling that what they have to say has not been heard or taken into account when coming to a decision - a form of token deliberation. Legitimacy and fairness require a comprehensive communication strategy that should map the context, identify target audiences, frame and portray the initiative and be explicit about the process. Processes that protect the agenda from political, economic and environmental shocks while still allowing room for change and adaptation (an "appeal mechanism" and a robust feedback loop) are important. This process of revision based on appeal must include explicit mechanisms for revising decisions based on emerging issues or arguments. It should include fora for alternative stakeholder viewpoints. Ideally the process should also include a dispute resolution mechanism that reduces hostility, is non-adversarial and that avoids 'winners and losers'. Walton et al [4] and Martin [3] have outlined a number of criteria to consider in a priority setting process:

1. **Documentation and legitimacy**: Was the priority setting process well documented, transparent and replicable? Was the decision making process clearly stated and decisions and the reasons for decisions broadly publicized? Was the priority setting process a fair and legitimate one? Does the method/s used include a process of compiling and using the best available information including that of national context (political, environmental, economic and health) allowing for a realistic priority setting process and eventual implementation?

2. **Stakeholder involvement**: Did the process involve the widest range of context-specific stakeholders?

3. **Revision/Appeals**: Was there a mechanism for revising decisions and was there a mechanism for dispute resolution?

4. **Leadership**: Were leaders responsible for ensuring that the first three elements are met, and were they responsible for monitoring, evaluating, and improving the decision making processes.

The objective of this paper is to test these criteria (framework) so as to establish the extent to which national priority setting processes incorporate these principles.

## Methods

Data were gathered from presentations made at the Geneva meeting and a search was conducted of Web of Science and PubMed for the period 2000-2008. Terms used in the search were "priority setting" AND "national" AND "level". Presenters at the Geneva meeting were also approached after the workshop for additional information. In many cases country experiences have not been well documented and as a consequence data were limited. These data were still included so as to provide a sense of the number of low and middle income countries that have engaged in some form of national priority setting.

## Results

Country presentations (and additional material from presenters) at the Geneva meeting were surveyed and analysed for the following countries - Malaysia, Cameroon, South Africa, Peru, Brazil and Argentina. The web search resulted in a total of 83 articles of which 67 were not relevant (not related to health or not about priority setting at all). Of the remaining 16 articles, 7 dealt with priority setting in rich countries; one article considered priority setting in a low and middle income country (Uganda) but at the district rather than national level; 5 were theoretical papers on priority setting; and one described the global response at national level to influenza pandemics. Table 1 presents summary details of the country experiences surveyed.

**Table 1:** Summary of country experiences

*COUNTRY*	*METHOD USED*	*DOCUMENTATION AND LEGITIMACY*	*STAKEHOLDER INVOLVEMENT*	*REVISION OR APPEALS PROCESS*	LEADERSHIP
**Malaysia**	Two approaches: Combined Approach Matrix (CAM) and identification of information gaps	This process involved prioritization by facilitators or experts of potential research topics. Priority Lists were reviewed and validated by a broader group of stakeholders and widely publicized	Selected groups of stakeholders involved a wide range of experts and health care managers from the public sector, private sector, professional organisations and academia.	No appeals process.	Ministry of Health

Cameroon	ENHR (Essential National Health Research) approach with support from COHRED.	ENHR approach with support of COHRED,-Ministry of Science and Technology, -List of research priorities, - endorsing and managing the agenda	Very limited stakeholder involvement- only Ministry of Science and Technology,	No appeals process. Stated as an objective but no plan	Single government department led

Peru	COHRED used as a reference	Researcher hired to develop research priorities. Two reports presented and discussed at a workshop.	Limited stakeholder representation	No appeals process.	Researcher led

South Africa (1)	ENHR approach	Following the ENHR priority setting process, single government department focuses on 12 sectors. Process used the Delphi method.	Some stakeholder representation	No appeals process	Single government department

South Africa (2)	Child Health and Nutrition Research Initiative (CHNRI)	Small group of technical experts	Medium sized group of stakeholders comprising professionals, members of the public.	No appeals process.	Researcher

Brazil	COHRED	The procedure comprised five well documented steps.	Priority research topics were submitted for public consultation. Extensive stakeholder involvement and public consultation. Transparent process with wide consultation	No appeals process.	Ministry of Health

Philippines	COHRED	Bottom-up approach with consultation at three levels: regional, zonal and national.	Poor stakeholder involvement. Not all participants considered the process relevant.	No appeals process.	Department of health and the Philippine Council for Health Research and Development

Pakistan	Combined Approach Matrix	The first step was the organization of a national seminar to develop priorities for health research. Participants included members from Health, Population Welfare, and Science and Technology Ministries, health academic institutions, university departments, the private sector and the NGO community.	No stakeholder involvement	No appeals process.	Ministry of Health

Argentina	Combined Matrix Approach	CAM used to guide financing strategies for the health research priorities identified.	No stakeholder involvement	No appeals process.	Researcher led with support from National Commission for Health Research and Ministry of Health

### 1. Method used

Methods used by the countries in their priority setting processes ranged from ones developed by the countries themselves to the use of existing methodologies. These included the Combined Matrix Approach CAM) [10]; the Council on Health Research and Development (COHRED) [11]; and the Child Health and Nutrition Research Initiative (CHNRI) [12]. Table 2 provides a summary of the three methodologies.

**Table 2:** Structured priority setting methodologies

Council on Health Research and Development (COHRED)	• Defines who sets priorities and how to get participants involved, the potential functions, roles and responsibilities of various stakeholders, information and criteria for setting priorities, strategies for implementation and indicators for evaluation
	• Specifies broad research avenues
Combined Approach Matrix (CAM)	• Systematic classification, organization and presentation of large body of information
	• Incorporates many dimensions
	• Recently included gender and poverty dimensions
	• Specifies broad research avenues
	• Identifies gaps in knowledge and future challenges
	• CAM can be applied at the level of disease, risk factor, group or condition, and also at local, national, or international level

Child Health Nutrition Research Initiative (CHNRI)	• Principles of legitimacy and fairness
	• Detailed listing of individual questions
	• Individual questions scored against pre-defined criteria. Technical experts independently score each research option
	• Stakeholder input is sought and used to provide relative weight of the criteria

### 2. Documentation and legitimacy

Legitimacy and fairness require a comprehensive communication strategy which ultimately facilitates transparency. This should map the context, identify target audiences, frame and portray the initiative, be explicit about the process, ensure the use of appropriate language, and identify the communication vehicles appropriate for target audiences. Countries varied markedly in the extent to which the priority setting processes were documented. Some countries such as Malaysia and Brazil have well documented processes. Malaysia was highly transparent and devoted a website to the priority setting process with specific details on how stakeholders were chosen, as well as the timetable of the process. One of the strengths of the Malaysia process is the fact it was the only exercise that has been repeated allowing for comparison across time to assess changing priorities. Brazil was successful in that it ensured transparency firstly, by guaranteeing a high level of public consultation, and secondly, by documenting processes which were accessible on the internet. In South Africa there was a measure of transparency in the scoring system in that the method facilitated the scores of individuals being available [13].

Outside these three countries there was poor transparency in terms of how the priority setting process was described, how members of the committee were chosen, or who they represented. Cameroon and Argentina for example, have poorly documented processes with difficult public access. In the case of Cameroon, the underlying rationale for priority setting and its importance are well articulated but there is no sense of the process, or any description of the priorities decided upon. In terms of legitimacy and fairness, the paucity of detail about the priority setting process and the lack of transparency was common across many of the countries.

### 2. Stakeholder involvement

The composition of stakeholder groups is normally considered to be country-specific, and includes groups such as government, funders, scientists, civil society, NGO's, non-scientist clinicians (physicians and nurses), health managers, academics, industry (broadly considered), patients and ethicists. The guiding principle in this process is one of inclusivity in order to ensure as wide participation as possible. Ongoing stakeholder deliberation should be encouraged, allowing for equitable voice, constructive debate and conflict resolution [14]. In the countries surveyed there is commonly no stakeholder involvement in a systematic and meaningful way. In some countries such as Peru there is no stakeholder involvement while in Cameroon stakeholder involvement is described as important but there is little evidence of this or whether mechanisms have been (or are being) put in place in order to ensure that it does happen. Malaysia on the other hand has a well developed system of inviting stakeholder input, mainly of experts and professionals involved in the public health system. However, there appears to be little involvement from user groups or members of the public, whereas Brazil appears to have significant stakeholder consultation by ensuring involvement in research priority topics by members of the public.

### 3. Revision and appeals

Processes that protect the agenda from political, economic and environmental shocks while still allowing room for change and adaptation (an "appeal mechanism" and a robust feedback loop) are important. This process of revision based on appeal must include explicit mechanisms for revising decisions based on emerging issues or arguments. It should include a platform for alternative stakeholder viewpoints. None of the countries surveyed had a systematic or operational appeals process to facilitate changing or rejecting particular decisions. The fact that the priority setting process in Malaysia has been repeated suggests that decisions made during previous rounds can be considered and revised based on changing needs. In most countries the priority setting process is a once off event.

### 4. Leadership

The two countries that appear to have the most well developed national priority setting mechanisms in place (Malaysia and Brazil), were also the two countries with the most senior levels of leadership guiding (and answerable) to the process. In both cases, priority setting was guided by the Health Ministries in the respective countries with senior representation throughout. In Malaysia, the priority lists were reviewed and validated by a broad group of stakeholders, while in Brazil the priority lists were submitted to a more limited group of stakeholders. In the case of countries where the process was led by researchers only one country (South Africa) approached stakeholders for their input.

## Discussion

This article outlines the priority setting processes of a selected group of low and middle income countries and measures these processes against a set of criteria. Malaysia and Brazil were the countries that fared best when their priority setting processes were judged against the criteria. Across all countries there was little evidence of how audiences were targeted and whether information fora were held in order to disseminate findings and appropriate knowledge. While many countries state the importance of dissemination and monitoring the implementation and impact of the agenda, there was little evidence of any mechanisms to ensure that this in fact happens. In addition, feedback to participants is in most cases limited. Linked to this was the frequent principle (across countries) of stating the importance of stakeholder engagement. Again, there was little evidence of the mechanisms for achieving this. The relative lack of genuine stakeholder engagement in priority setting processes in surveyed countries has implications for whether there has been a consideration of the widest range of relevant values [3]. Ultimately, this compromises the 'buy in' of stakeholders.

Both Brazil and Malaysia had high level government involvement in the process. This was not the case in many of the other countries. High level leadership is central if national level priority setting processes are going to prove transformational. The overall leadership structure may vary between countries, may be designated in law, and may involve collaboration between different ministries. The leader/s of the priority setting initiative should be respected within the research community, have the relevant experience and have credibility amongst constituents. Furthermore, as part of ensuring political buy in, space should be created for government leaders (such as, for example, the Minister of Health, Minister of Finance, Minister of Science and Technology, Prime Minister etc) to suggest priorities and to feel that they are part of the priority setting process. This is also important in terms of revisions and appeals as a greater number of champions within the political arena will ensure that processes are transparent and that changes in any one party will not render the entire process vulnerable.

According to Kapiriri and colleagues [9] fair priority setting requires that four conditions are met - relevance, publicity, revisions and enforcement. In none of the countries surveyed were the conditions of revision and enforcement met. In no country was there an effective appeals process. The 'rolling 5-year plan' of Malaysia came closest to an active appeals process. One of the consequences of this is that opportunities allowing for equitable voice, constructive debate and conflict resolution did not exist. For example, despite their significant stakeholder involvement, the priority setting process that Brazil engaged in is quite instructive.

Experiences from countries that have engaged in priority setting exercises indicate that a number of guiding principles may be helpful in ensuring balance. One consideration is to intentionally include only individuals with *diverse and relevant experiences *and viewpoints as opposed to including representatives from a variety of societies and associations. In the interests of agreement it may be beneficial to have some stakeholders as ex officio participants in order that they can contribute their views but are not involved in actual decision making. Another useful strategy is the establishment of a communication channel with neighboring countries about the priority setting process. This serves as a gesture of goodwill, but may also aid the priority setting process. This is pertinent in the case of countries without comprehensive Burden of Disease data, but where neighboring countries may have relevant (similar) data.

With regard to the *monitoring and evaluation *of the priority setting process the guiding principle should fit in with the framework of legitimacy and fairness. The monitoring and evaluation plan should take into consideration the processes, outcomes and impacts including the values established by stakeholders. So while in Peru there was a recommendation to create a National Health Research Committee to monitor research and guide specific research agendas this has not been implemented. Peer review mechanisms and tools need to be developed to support monitoring and evaluation, and there needs to be investment in robust health systems, including health information systems and national health accounts that can collect reliable information for informed decision making. The monitoring and evaluation framework should also include a framework that includes stakeholder perceptions of the process which identifies strategies for improving the process.

The expected outcome of the *methodology or tool application needs to be explicitly stated *from the outset. Countries should consider a strategy that allows for tracking of direct and indirect impacts of resource allocation. Elements may include questions around what research has been done, what were the research findings, what policy or system changes were influenced by the research, and what was the overall effect of the prioritization? In the context of the inclusivity that is being highlighted by the priority setting process, accountability is critical and including tangible outcomes designed specifically to ensure accountability must be included.

In terms of networks, these would ideally be comprised of researchers in the public and private sectors, decision-makers in governments, and civil society. Most importantly, the very act of priority setting can provide valuable direction for the allocation of public and private research funds into areas of strategic importance. It can also serve to strengthen the role of national stakeholders as stewards of the national research agenda.

## Conclusions and recommendations

With more than $130 billion being invested globally into health research each year, setting priorities in health research investment at all levels is an increasing phenomenon. The lack of stakeholder involvement was a common finding in the survey. Countries have used a variety of approaches to include different stakeholders. One consideration is to intentionally include only individuals with diverse and relevant experiences and viewpoints as opposed to including representatives from a variety of societies and associations. An approach that commonly works well is to establish a small Executive Committee that guides the process and decision making; while a larger decision making group (comprised of stakeholders) would then be charged with implementing the chosen methodology and to make decisions. An Advisory Council comprising a much larger number of stakeholders (possibly separated into smaller groups) might also be created in order to advise, deliberate, provide viewpoints, and to provide support to the smaller decision making groups. Another useful strategy is the establishment of a communication channel with neighboring countries about the priority setting process. This serves as a gesture of goodwill, but may also aid the priority setting process.

An effective priority setting process for health research should take into account the Paris Declaration on Aid Effectiveness which is a broad international consensus about how to make aid more effective, while at the same time providing assistance to countries to develop their priorities and development plans [15]. Following the principles of harmonization and alignment with regard to a national health policy framework will help to facilitate consistency, synergy and effective implementation. It will also encourage broader external cooperation by potential donors and relevant stakeholders.

A strategy to advocate and disseminate appropriate knowledge - throughout the life of the process - would serve several objectives such as sharing of information, encouraging accountability, supporting a system for tracking, providing a platform for debate and attracting the research community. Priority setting is an iterative process, where each step of the process, each evaluation and feedback loop improves on the earlier one. Priority setting processes must be repeated, and previous rounds considered in later iterations. Priority setting processes must have in-built mechanisms for publicizing results, effective procedures to enforce decisions, establish an appeal process as well as processes to ensure that the revision of priorities happens in practice.

## Competing interests

The authors declare that they have no competing interests.

## Authors' contributions

MT attended the consultative workshop and has taken a major role in writing the submitted paper and approved the final version. MC attended the consultative workshop and has taken a major role in writing the submitted paper and approved the final version. NH conducted the literature search and has taken a major role in writing the submitted paper and approved the final version. IR attended the consultative workshop and has taken a major role in writing the submitted paper and approved the final version.

## References

[B1] Rudan I Chopra M Kapiriri L Gibson J Ann Lansang M Carneiro I Ameratunga S Tsai AC Chan KY Tomlinson M Hess SY Campbell H El Arifeen S Black RE Setting priorities in global health research investments: Universal challenges and conceptual framework Croatian Medical Journal 2008 49 307 317 10.3325/cmj.2008.3.307 18581609 PMC2443616

[B2] Alliance for Health Policy and Systems Research Sound choices: Enhancing capacity for evidence-informed health policy 2007 Geneva: Alliance for Health Policy and Systems Research/World Health Organization

[B3] Martin DK Stakeholder engagement in priority setting World Health Organization Consultative Workshop. Geneva, April 10-11, 2008. World Health Organization: Geneva

[B4] Walton NA Martin DK Peter EH Pringle DM Singer PA Priority setting and cardiac surgery: A qualitative case study Health Policy 2007 80 444 458 10.1016/j.healthpol.2006.05.004 16757057

[B5] Daniels N Accountability for reasonableness: Establishing a fair process for priority setting is easier than agreeing on principles British Medical Journal 2000 321 1300 1301 10.1136/bmj.321.7272.1300 11090498 PMC1119050

[B6] Daniels N Sabin J Limits to health care: fair procedures, democratic deliberation and the legitimacy problem for insurers Philosophy and Public Affairs 1997 26 303 350 10.1111/j.1088-4963.1997.tb00082.x 11660435

[B7] Singer PA Martin DK Giacomini M Purdy L Priority setting for new technologies in medicine: qualitative case study British Medical Journal 2000 321 1316 1318 10.1136/bmj.321.7272.1316 11090513 PMC27534

[B8] Bruni RA Laupacis A Martin DK Public engagement in setting priorities in health care Canadian Medical Association Journal 2008 179 15 18 10.1503/cmaj.071656 18591516 PMC2464477

[B9] Kapiriri L Tomlinson M Gibson J Chopra M El Arifeen S Black RE Rudan I Setting priorities in global child health research: Addressing the value of stakeholders Croatian Medical Journal 2007 48 618 627 17948948 PMC2213572

[B10] Ghaffar A de Francisco A Matlin S editors The combined approach matrix: a priority-setting tool for health research 2004 Geneva: Global Forum for Health Research

[B11] The Working Group on Priority Setting Priority setting for health research: lessons from developing countries Health Policy and Planning 2000 15 130 136 10.1093/heapol/15.2.130 10837035

[B12] Rudan I Gibson JL Ameratunga S El Arifeen S Bhutta ZA Black M Black RE Brown KH Campbell H Carneiro I Chan KY Chandramohan D Chopra M Cousens S Darmstadt GL Meeks Gardner J Hess SY Hyder AA Kapiriri L Kosek M Lanata CF Lansang MA Lawn J Tomlinson M Tsai AC Webster J Setting Priorities in Global Child Health Research Investments: Guidelines for implementation of the CHNRI Method Croatian Medical Journal 2008 49 720 733 10.3325/cmj.2008.49.720 19090596 PMC2621022

[B13] Tomlinson M Chopra M Sanders D Bradshaw D Hendricks M Greenfield D Black RE El Arifeen S Rudan I Setting priorities in child health research investments for South Africa PLoS Medicine 2007 4 8 e259 10.1371/journal.pmed.0040259 17760497 PMC1952202

[B14] Kapiriri L Norheim OF Martin DK Fairness, accountability for reasonableness. Do the views of priority setting decision makers differ across health systems and levels of decision making? Social Science and Medicine 2008 68 766 773 19070414 10.1016/j.socscimed.2008.11.011

[B15] Organisation for Economic Cooperation and Development The Paris Declaration on Aid Effectiveness http://www.oecd.org/dataoecd/11/41/34428351.pdf (accessed Dec 12, 2009)

